# Distinctive Glial and Neuronal Interfacing on Nanocrystalline Diamond

**DOI:** 10.1371/journal.pone.0092562

**Published:** 2014-03-24

**Authors:** Amel Bendali, Charles Agnès, Simone Meffert, Valérie Forster, Alexandre Bongrain, Jean-Charles Arnault, José-Alain Sahel, Andreas Offenhäusser, Philippe Bergonzo, Serge Picaud

**Affiliations:** 1 INSERM U968, Institut de la Vision, Paris, France; 2 Sorbonne Universités, UPMC Univ Paris 06 UMR_S968, Institut de la Vision, Paris, France; 3 CNRS UMR7210, Institut de la Vision, Paris, France; 4 CEA-LIST, Diamond Sensors Laboratory, Saclay, Gif-sur-Yvette, France; 5 Institute of Bio- & Nanosystems - Bioelectronics (IBN2) Forschungszentrum, Juelich, Germany; 6 Centre Hospitalier National d'Ophtalmologie des Quinze-Vingts, Paris, France; 7 Fondation Ophtalmologique Adolphe de Rothschild, Paris, France; 8 Institute of Ophthalmology, University College of London, London, United Kingdom; 9 French Academy of Sciences, Paris, France; Université de Technologie de Compiègne, France

## Abstract

Direct electrode/neuron interfacing is a key challenge to achieve high resolution of neuronal stimulation required for visual prostheses. Neuronal interfacing on biomaterials commonly requires the presence of glial cells and/or protein coating. Nanocrystalline diamond is a highly mechanically stable biomaterial with a remarkably large potential window for the electrical stimulation of tissues. Using adult retinal cell cultures from rats, we found that glial cells and retinal neurons grew equally well on glass and nanocrystalline diamond. The use of a protein coating increased cell survival, particularly for glial cells. However, bipolar neurons appeared to grow even in direct contact with bare diamond. We investigated whether the presence of glial cells contributed to this direct neuron/diamond interface, by using purified adult retinal ganglion cells to seed diamond and glass surfaces with and without protein coatings. Surprisingly, these fully differentiated spiking neurons survived better on nanocrystalline diamond without any protein coating. This greater survival was indicated by larger cell numbers and the presence of longer neurites. When a protein pattern was drawn on diamond, neurons did not grow preferentially on the coated area, by contrast to their behavior on a patterned glass. This study highlights the interesting biocompatibility properties of nanocrystalline diamond, allowing direct neuronal interfacing, whereas a protein coating was required for glial cell growth.

## Introduction

The development of neuroprostheses and brain-machine interfaces has increased exponentially following the success of cochlear implants and deep brain stimulation for Parkinsonian patients [1]. The concept of visual prostheses was recently validated in clinical trials, which showed that they could enable blind patients to read short words, to identify contrasting objects or to follow a lane on the ground [2], [3], [4]. Retinal prostheses were introduced into the subretinal space (subretinal implants) or fixed to the vitreal side of the retina (epiretinal implant). Subretinal implants stimulate retinal bipolar cells, neurons normally postsynaptic to photoreceptors [3], whereas epiretinal implants target retinal ganglion cells (RGCs), spiking neurons sending visual information to higher visual centers [2]. If this link to higher visual centers is lost, visual prostheses must be implanted within or on higher visual centers [4]. Such implants have already restored some visual function in patients [2], [3], [4], but larger numbers of electrodes and greater individual electrode resolution are required for the performance of more complex tasks, such as face recognition, text reading or independent locomotion. Various three-dimensional (3 D) implant designs involving pillars [5] or wells [6] have already been put forward.

Alternatively, resolution could be achieved by direct neuron/material interfacing. Indeed, the implant is often in contact with a surface glial layer, as in the retinal implants described above. Penetrating electrodes have been used in attempts to resolve this problem, but the glial cells grow around them [7], thereby altering the neuron/electrode interface, leading to a loss of function after a few months [8]. New electrode materials displaying positive adhesion to neurons and repulsing glial cells are therefore required. Nanocrystalline diamond has recently been identified as potentially useful for neuroprosthesis applications, because boron-doped diamond has semiconductor properties [9], [10], [11], [12]. Such form of synthetic diamond is grown in the form of coatings with grain sizes in the 10–30 nm range. Its advantages as an electrode material have already been assessed for cell recording [13] and for the stimulation of retinal ganglion cell neurons [14]. Diamond has also been shown to be biocompatible with osteoblast cultures [15], with neural stem cells [16], [17] and with embryonic cortical cell cultures [18], [19]. In fact, a particular form of such synthetic diamond layers, so called ultra-nanocrystalline diamond since it exhibits a very small average grain size (in the 5–10 nm range), was even found to promote proliferation and differentiation of neural stem cells into neurospheres [16], [17]. However, for biocompatibility studies, this cell proliferation of neural stem cells could mask a material toxicity. Furthermore, the required addition of neurotrophic factors to their culture could prevent neuronal degeneration and thus mask a potential material toxicity. Diamond biocompatibility was also assessed in monolayer cell cultures with differentiated embryonic neurons. These embryonic neuronal cells were found not to survive directly in contact with either polycrystalline or nanocrystalline diamond layers [18], [19]. However, they could grow on a protein pattern stamped on polycrystalline diamond [18] or on protein-coated nanocrystalline diamond [19]. Surprisingly, nanodiamond particles dispersed on the nanocrystalline diamond also promoted the embryonic cell survival [19]. However, such dispersed nanodiamond particles are unlikely to be acceptable for *in vivo* applications, due to the risk of mobility within the body. Finally, the presence of glial cells in embryonic and stem cell cultures complicates the assessment of direct diamond/neuron interfacing.

In this study, we addressed the biocompatibility of nanocrystalline diamond layers, using retinal cells or purified RGC neurons from both adult and postnatal animals.

## Materials and Methods

### Diamond growth and analyses

Nanocrystalline diamond films were produced as previously described [10], [20] using the microwave enhanced chemical vapor deposition technique (MWCVD) from methane and hydrogen mixtures. After growth, the layers as naturally Hydrogen-terminated were stored in a sterile Petri dish on their removal from the reactor and were rinsed with sterile culture medium before cell seeding. We avoided the use of oven sterilization in all cases. Diamond oxidation was achieved by immersion for 30 minutes in a boiling solution containing 98% concentrated sulfuric acid and excess potassium nitrate (H_2_SO_4_/KNO_3_).

A UV-Vis Spectroscopic Ellipsometer (Horiba Jobin-Yvon, UVISEL), at a fixed angle of 70°, was used to characterize the roughness and thickness of the diamond films before and after incubation (45 min, room temperature) with pure deionized water or water containing 0.1 mg/ml poly-D-lysine (PDL) (Sigma-Aldrich). The films were then rinsed in water and dried under argon. We used a Tauc-Laurentz dispersion law [21] to determine the optical constants. The same three-layer microstructural model taking into account surface roughness/diamond/silicon [20] was fitted to the data, to simplify the system: the measured variation in diamond thickness provides a semiquantitative representation of the thickness of the PDL layer deposited on the diamond surface.

In X-ray photoelectron spectroscopy (XPS), binding energies were expressed relative to the Au4f_7/2_ peak located at 83.6 eV. A curve fitting procedure was applied to smoothed spectra, to extract the components in the N1s spectrum, using Voigt functions with Lorentzian half-widths of 0.2 eV and 0.6 eV.

### Primary retinal cell cultures, viability and quantification

All experiments were carried out in accordance with European Community Council Directives (86/609/EEC) and with the ARVO (Association for Research in Vision and Ophthalmology) statement for the use of animals in ophthalmic and visual research. Ethics committee approval is not required for cell culture experiments, as stated in European Union Directives (2010/63/UE). Animals were killed by CO_2_ sedation and cervical elongation, and all efforts were made to minimize suffering.

Mixed retinal cell cultures and purified retinal ganglion cells (RGCs) were prepared from adult and postnatal Long Evans rats (Janvier, Le Genest Saint-Isle, France), as previously described [22], [23]. Glass coverslips or diamond samples were coated uniformly with protein by incubating successively with poly-D-lysine (2 μg/cm^2^, 45 min) then laminin (1 μg/cm^2^ overnight) (Sigma-Aldrich). For protein patterns, microstamps were produced by photolithography and molding [24], [25]. They were immersed for 20 min in 10 μg/ml FITC (fluorescein isothiocyanate)-conjugated poly-L-lysine (FITC-PLL) mixed with 2 μg/ml laminin, both diluted in Hank's balanced salt solution. The stamp was dried in a stream of nitrogen and pressed onto the diamond substrate for two minutes.

Mixed adult cells were used to seed the substrate at a density of 2×10^5^ cells/cm^2^ in Neurobasal-A medium containing L-glutamine (2 mM) and B27 (2%) (Life Technologies). Pure adult and postnatal RGCs were used to seed substrates at a density of 2×10^4^ cells/cm^2^ in the same NBA+ medium or a ND-G medium, respectively. Cell viability was indicated by calcein green fluorescence following incubation with the “live-dead” test kit (Life Technologies) in phosphate buffer saline (PBS, Sigma-Aldrich) for one hour in a humidified chamber (37°C, 5% CO_2_). RGC survival was calculated by normalizing the density of calcein AM-positive cells at 6 days *in vitro* (DIV) by that measured at 1 DIV in the same experiment. Neurite lengths and colocalization on the protein patterns were quantified with MetaMorph software (Roper Scientific). For the control conditions in the quantification of pattern colocalization, a virtual grid was superimposed to the images of cells on the unpatterned materials in order to calculate a theoretical colocalization percentage for these control conditions. Cells were identified after fixation (4% paraformaldehyde (Sigma) in PBS, 15 min, room temperature), by immunolabeling with primary anti-G0α (mouse monoclonal, dilution 1∶1000, Chemicon) and anti-GFAP (rabbit polyclonal, dilution 1∶500, Dako) antibodies in mixed culture, and with anti-NF200 primary antibody (rabbit polyclonal, dilution 1∶1000, Sigma) for purified RGCs on protein patterns. Cell quantification was performed automatically with the Metamorph software. Glial cell counts were estimated by surface coverage whereas bipolar cells were counted individually with a 5 μm-diameter threshold and an additional circular criterion to avoid cell debris.

## Results

**Figure 1:**
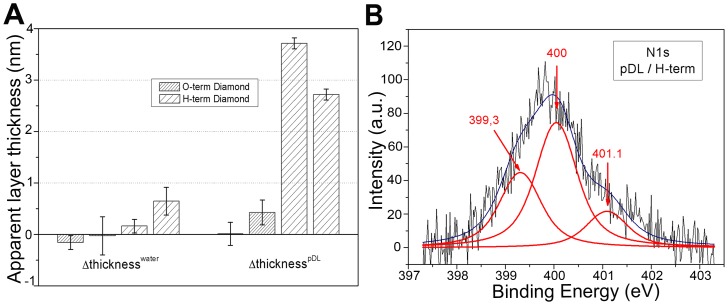
Characterization of the poly-D-lysine coating on diamond. (**A**) Ellipsometry measurements on two H-terminated and two O-terminated diamond samples. Variation of apparent diamond thickness (nm) measured by ellipsometry after 45 minutes of incubation with water or poly-D-lysine (pDL). It seems to be possible to generate a measurable poly-D-lysine coating on hydrogen-terminated diamond surfaces, but not on oxygen-terminated diamond surfaces. (**B**) XPS spectrum on hydrogen-terminated diamond surface. N1s core level of poly-D-lysine coated H-terminated diamond. Data are expressed as means ± SEM from 3 experiments.

Neuron survival in culture requires the substrate considered to be coated with proteins or extracellular matrix proteins before cell seeding [26], [27], [28], [29]. We therefore investigated whether protein coatings would adhere to nanocrystalline diamond. Diamond samples with different surface terminations were incubated in a poly-lysine solution and characterized by ellipsometry and X-ray photoelectron spectroscopy (XPS). We compared two types of diamond sample: hydrogen-terminated (as grown) and oxygen-terminated (chemically treated). These diamond surfaces were prepared uncoated or were coated with poly-D-lysine only, as the charges deposited by poly-lysine facilitate laminin adhesion in the second step of the coating procedure (see Methods). The presence of poly-lysine therefore provides a direct indication of the likelihood of a protein adhering to these diamond surfaces. Figure 1a illustrates, for two samples, the variation of the apparent thickness of the layer, measured by ellipsometry after a 45-minute incubation with either water or poly-D-lysine, for both H-terminated and O-terminated diamond samples. On O-terminated diamond samples, a very small variation (<0.5 nm) of the apparent thickness of the layer was observed after incubation with poly-D-lysine or water. By contrast, on H-terminated diamonds, a 3 nm variation of layer thickness was observed after 45 min of poly-D-lysine incubation, whereas little or no variation was observed after incubation with water. Thus, a poly-D-lysine coating was clearly formed on H-terminated diamond surfaces, whereas this coating was thinner on O-terminated diamond surfaces.

We characterized the chemical nature of the protein coating on diamonds (nitrogen atoms of poly-D-lysine) further, by performing X-ray photoelectron spectroscopy (XPS). The survey spectra revealed that carbon, oxygen and nitrogen were the predominant elements on the coated H-terminated diamond. As no nitrogen was observed on uncoated H-terminated diamond or on coated and uncoated O-terminated diamond, we studied core nitrogen levels to characterize the protein coating. Figure 1b shows the core nitrogen (N1s) spectrum after 45 minutes of incubation with an H-terminated diamond sample. This large peak, centered at 400 eV, can be deconvoluted into three components at 399.3, 400 and 401.1 eV. These components have been attributed to deprotonated amine (-NH_2_) [30], amide (-NCO-) [31] and protonated amine (-NH_3_^+^) [32], respectively. The detection of these components further confirmed the presence of a poly-D-lysine coating on the coated H-terminated diamond surface.

**Figure 2:**
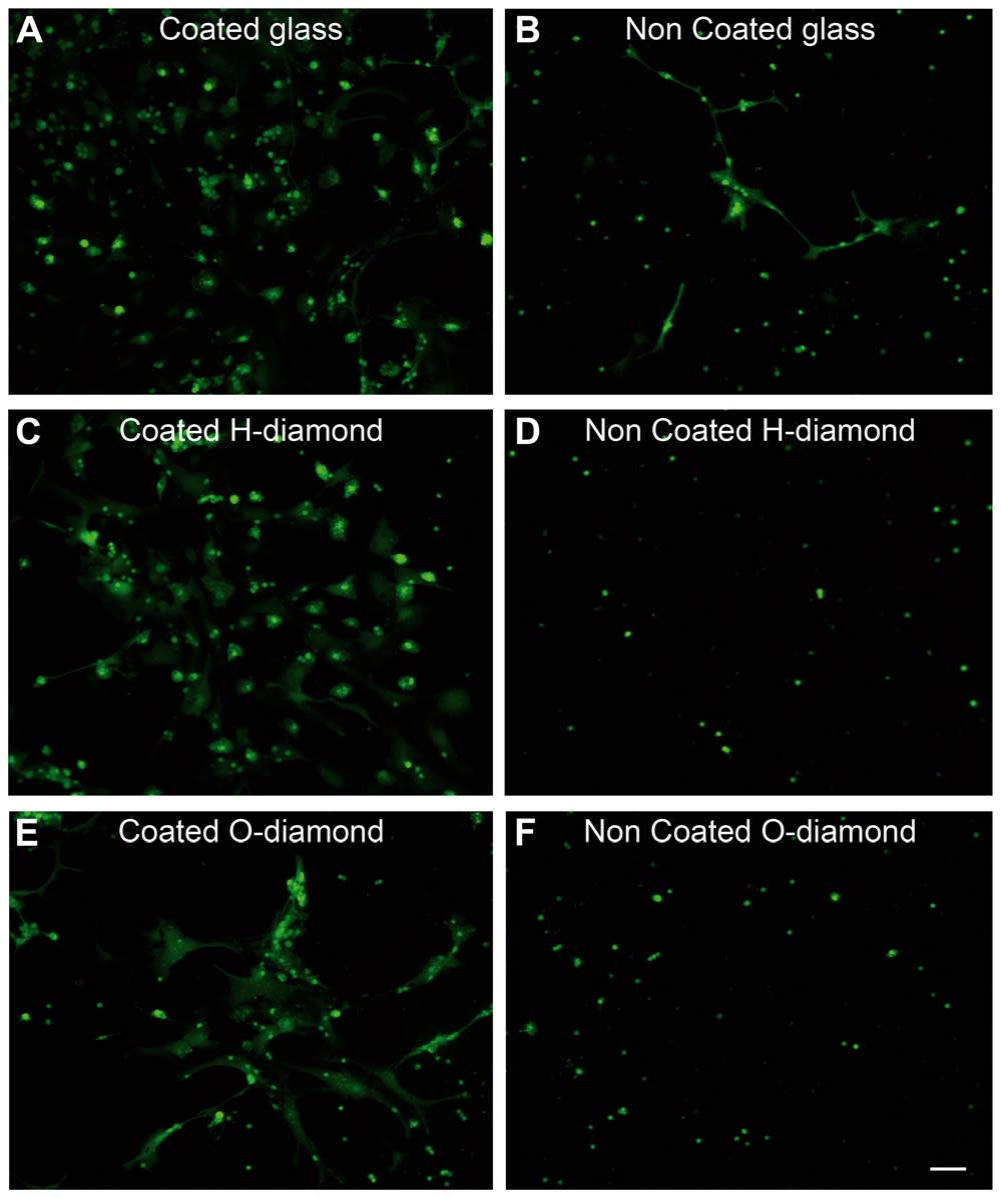
Viability of mixed neurons and glial cells on diamond. (**A**) protein-coated glass, (**B**) bare glass, (**C**) protein-coated H-terminated diamond, (**D**) bare H-terminated diamond, (**E**) protein-coated O-terminated diamond, (**F**) bare O-terminated diamond. Note the presence of macroglial cells on the coated substrates (**A**, **C**, **E**) whereas small neuronal cells are visible on all substrates. The scale bar represents 50 μm.

We then investigated cell survival on diamond, initially with mixed retinal cell cultures on two diamonds with the two different terminations: hydrogen-terminated diamond and chemically oxidized diamonds. Retinal neurons have also been reported to require a protein coating to adhere and survive [26]. We therefore examined cell survival on glass and diamond with and without a protein coating (poly-D-lysine and laminin). Cell viability was first checked on all substrates, with the “live/dead” assay. Figure 2 illustrates mixed retinal cell cultures on coated (Fig. 2A) and uncoated glass (Fig. 2B) when cells are revealed by a vital dye (Calcein-Ethidium Homodimer, Life Technologies). On the uncoated glass, one small cell population can be seen with occasional neurites whereas a second larger cell type can be seen on the coated glass. On the coated H-terminated diamond substrates, both cell types can clearly be observed (Fig. 2C) whereas no or very few cells subsisted after 10 days in vitro on the uncoated H-terminated diamond (Fig. 2D). This result indicated that H-terminated diamond was not supporting cell survival. However, some variability was observed in the rate of survival on such uncoated H-terminated diamond. On the coated O-terminated diamond (Fig. 2E), essentially fewer cells, including large cells, were present. The culture on the uncoated O-terminated diamond surfaces (Fig. 2F) appeared similar although leaving mainly small cells suggesting thereby that the cell survival on O-terminated diamond was less influenced by the coating. These cultures indicated that different cell types can survive on coated H-terminated diamond whereas mainly only one cell type can grow on uncoated O-terminated diamond surfaces.

**Figure 3:**
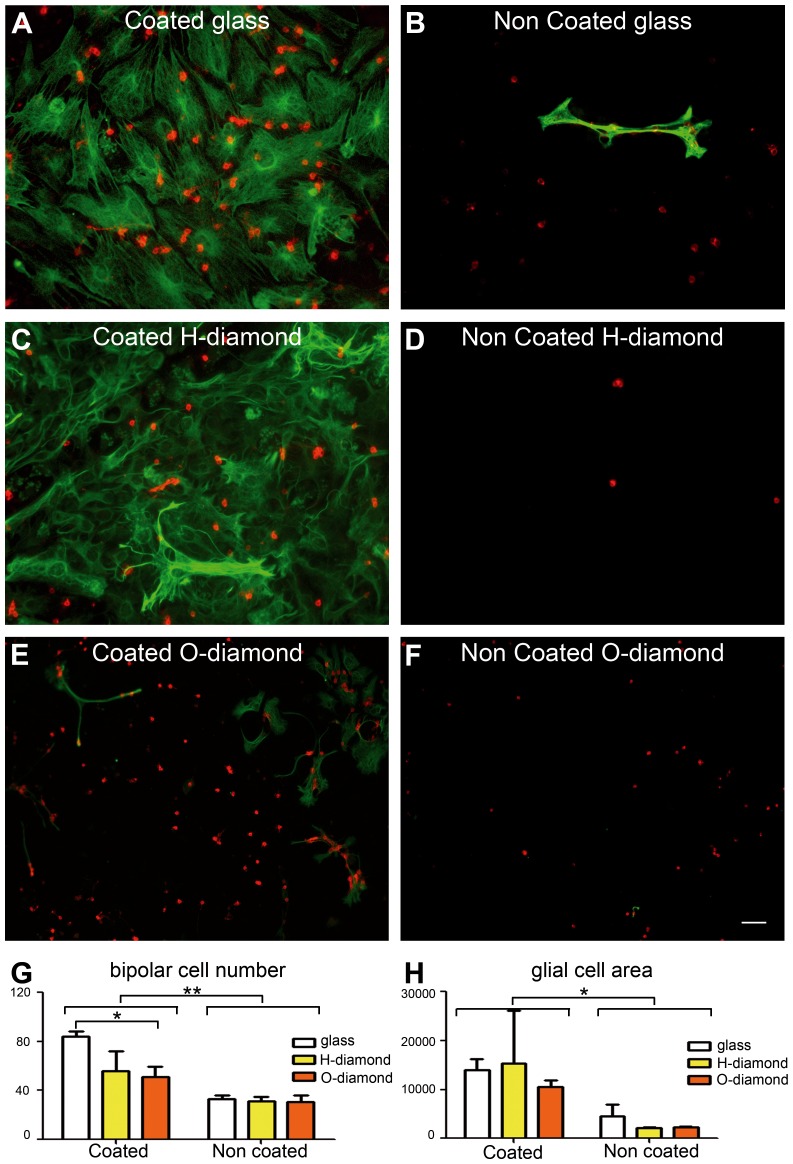
Survival of mixed neurons and glial cells on diamond. (**A**) protein-coated glass, (**B**) bare glass, (**C**) protein-coated H-terminated diamond, (**D**) bare H-terminated diamond, (**E**) protein-coated O-terminated diamond, (**F**) bare O-terminated diamond. Glial cells and bipolar cells were identified, with an anti-GFAP (green) and an anti-Goα (red) antibody, respectively. (G-H) Quantification of the neuronal bipolar cells (G) and glial cells (H: ×10^3^ μm^2^). (means ± SEM, *n* = 4 experiments, 3 samples/group/experiment). Two-way ANOVA was carried out, followed by a Bonferroni post-hoc test (**p<0.01, *p<0.05). The scale bar represents 50 μm.

To further assess the nature of these surviving cells on diamond, different cell markers were applied on the cultures. We determined which cells survived by immunolabeling for glial cells (GFAP: green in Fig. 3) and for neurons, such as ON bipolar neuronal cells (G0α: red in Fig. 3). Glial cells were highly abundant on the coated glass and coated H-terminated diamond surfaces (Fig. 3D), but were less widespread on the coated O-terminated diamond samples (Fig. 3A, D). Very few glial cells were detected on any of the uncoated substrates (Fig. 3B, D). These observations were confirmed by measuring glial cell surface coverage in all conditions (Fig. 2D), although substantial between-experiment variability was observed for coated H-terminated diamonds. Due to this variability, we used only O-terminated diamonds for subsequent studies.

Bipolar neuronal cells survived in all conditions, even in direct contact with the substrates (Fig. 3A–C). Indeed, bipolar cells were present on uncoated diamond, despite the very small number of glial cells on this type of substrate (Fig. 2B–D). Similarly, they were also present on the uncoated glass, contrary to what has classically been reported (Fig.3C). Indeed, bipolar cell survival appeared to be lower on coated diamonds than on coated glass (Fig. 3C). These results suggest that glial cells prefer protein-coated substrates, whereas neurons can survive on uncoated substrates, such as diamond.

**Figure 4:**
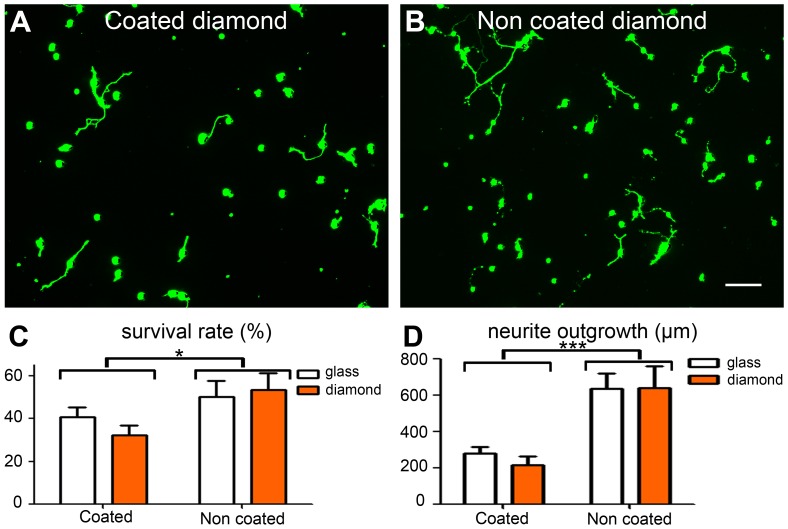
Survival of pure adult retinal ganglion cell neurons on O-terminated diamond. (**A**) Protein-coated diamond, (**B**) bare diamond. (**C**) Quantification of viable retinal ganglion cells at 6 days *in vitro*, normalized with respect to the number of these cells on day 1 *in vitro*. (**D**) Quantification of neurite length at 6 days *in vitro* (means ± SEM from *n* = 4 experiments, 3 samples/group/experiment). Two-way ANOVA was performed, followed by a Bonferroni post-hoc test (***p<0.001, *p<0.05). The scale bar represents 50 μm.

We assessed neuronal survival on diamond further and investigated the behavior of RGCs on diamond, using purified adult RGCs [23]. These spiking neurons were chosen for study because they are very rare in mixed adult retinal cell cultures (less than 1%). Their quantification in mixed retinal cell cultures would, therefore, not be meaningful. Furthermore, the use of purified adult RGC preparations made it possible to seed the surface of the diamond with purified adult spiking neurons in the absence of glial cells. In “live/dead” tests, the morphology of these cells appeared to be very similar in all conditions, whether on glass or diamond, with or without coating (Fig. 4A–B). We quantified the cell bodies and normalized the value obtained with the survival rate on day 1. Only the differences between the coated and uncoated substrates were significant (Fig. 4C). Pure RGCs had longer neurites on uncoated substrates, and the differences between coated and uncoated diamond and between coated and uncoated glass (Fig. 4D) were statistically significant (*p*<0.001). Thus, isolated adult RGCs can survive on diamond, but the growth of their neurites is reduced by coating the surface with a protein.

**Figure 5:**
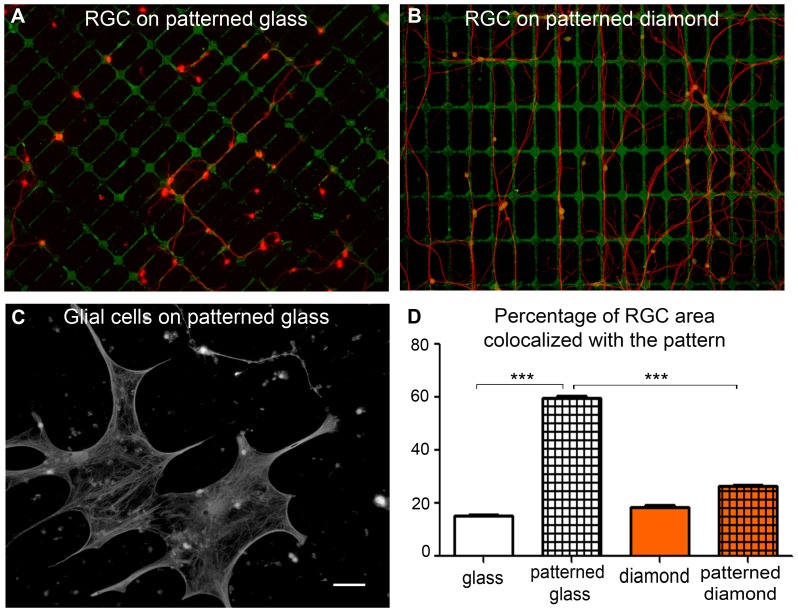
Distinct glial and neuronal cell preference for diamond protein-patterned substrates. Pure postnatal (p7) retinal ganglion cells (RGCs) on a protein-patterned glass (**A**) or protein-patterned diamond (**B**) substrate. RGCs were identified on the basis of anti-NF200 immunolabeling (red fluorescence) on the protein pattern (green) (**C**). Mixed retinal cell culture showing the preference of the glial cells for the protein pattern on glass. (**D**) Quantification of the protein pattern surfaces on glass and diamond, with the overlap between RGCs and these protein patterns. (means ± SEM, *n* = 4 experiments). Two-way ANOVA was performed, followed by a Dunn's post-hoc test (***p<0.001). The scale bar represents 50 μm.

Unlike embryonic cortical neurons [18], [27], [28], [29], adult RGCs appeared to prefer uncoated substrates for neurite outgrowth. A preference for protein-coated diamond was previously reported in a study using patterned diamond substrates [18]. We therefore provided retinal neurons with the same alternatives, by using them to seed diamonds with a patterned protein coating. The glial cells stretched out between the lines of proteins in the patterns, confirming the strong preference of these cells for protein-coated glass (Figure 5C). Many neurons survived above the glial cells. This made it difficult to determine, with these mixed retinal cells, the behavior of retinal neurons with respect to the protein patterning. We addressed this issue independently of glial cells, by generating pure postnatal RGC cultures on the same protein patterns, first on glass and then on diamond. Postnatal RGCs were used because they grow long neurites in a few days. On glass, these postnatal RGCs followed the shape of the rectangular protein patterns (Fig. 5A) giving the cultures an organized structure not seen in control conditions. When the protein pattern was superimposed onto the cells, it became obvious that the RGC neurites followed the pattern (Fig. 5A). The areas of the RGC processes overlapping the pattern (60%±1, *n* = 3) were then quantified automatically, and were clearly different from the substrate areas occupied by the protein pattern (15%) (Fig. 5D). This indicates that RGC neurons prefer the protein coating on glass. This preference for the protein coating is surprising in view of the increased RGC survival and growth on uncoated glass (Fig.4), the difference could result from protein stamping (Fig.5) instead of protein bath application (Fig.4) or from the use of postnatal cells instead of adult cells. In a similar experiment on diamond, some neurites followed the protein patterns, but most were observed on the uncoated diamond (Fig. 5B). The fraction of neurites overlapping the pattern (26%) approached the proportion of the area covered by the pattern (18%). This value differed significantly (*p*<0.001) from that obtained on glass (Fig. 5D). Thus, RGCs display no major preference for the protein coating on diamond, by contrast to our findings for glass.

## Discussion

Diamond is, potentially, an excellent candidate biomaterial for use in neuroprosthesis electrodes, because it is highly mechanically stable and has semiconductor properties when doped with boron, providing a large electrochemical potential window for electrical stimulations. The use of nanocrystalline diamond for such medical applications will require evidence of its biocompatibility for neuronal tissues. Several studies addressed this question with cultures of embryonic cortical neuronal cells [18], [19] or neural stem cells [16], [17]. For stem cells, neurosphere development on ultra-nanocrystalline diamond was attributed to cell adherence following protein adsorption onto the material [17]. In monolayer cell cultures, polished polycrystalline or nanocrystalline diamonds appeared to provide substrates equivalent to glass for neuronal cell attachment and growth. Indeed, neuronal growth on all these surfaces was observed only if the substrate was coated with a protein [18], [19] or with dispersed nanodiamond particles [19]. The embryonic cells used in these studies therefore behaved differently from our adult or postnatal retinal neurons. Indeed, we found that both retinal bipolar neurons and RGC neurons grew in direct contact to nanocrystalline diamond. Using purified adult RGCs, we even showed that these spiking neurons developed more effectively on an uncoated diamond than on the protein-coated diamond. Furthermore, postnatal RGCs did not follow the protein pattern stamped on diamond when provided with a choice, whereas embryonic neurons did so in a previous study [18]. However, there are major differences between this previous study [18] and our investigation. First, different tissues were used: retina in our work and cortex in the previous study. Second, different developmental stages were used for cell preparation: neural stem cells and embryonic cells were used in previous studies, whereas we used adult and postnatal neurons. Our experimental design, with the use of adult neurons, demonstrated that glial cell support [19], [23] was not required to achieve a direct neuron/diamond interface. The neuronal preference for uncoated diamond and uncoated materials provided a clear distinctive feature in cell adherence and survival with respect to glial cells. In the field of neuroprostheses, the creation of such a direct interface between neurons and diamond would greatly increase the efficacy and resolution of the electrodes. Such an interface could form if penetrating electrodes were used as in the case of the Utah arrays [7]. Because, gliosis around penetrating electrodes limits their long-term efficacy, the use of uncoated O-terminated diamond at the tip of penetrating electrodes could facilitate the direct neuron/electrode interaction while preventing the glial growth on the electrodes. This limitation of the glial reaction would increase the long-term efficacy of implants by maintaining the direct neuron/electrode interface.

Previous studies have shown that glial cells especially retinal Müller glial cells grow best on protein-coated substrates, in particular with laminin [33], [34] because the receptor to laminin seems much more expressed in glial cells for their fixation to the basal membrane and blood vessels. Our results confirmed these findings, as cell density was lower on uncoated glass and on uncoated diamond than on their protein-coated counterparts. Further evidence of this preference was provided by the square shapes of the glial cells on the protein patterns, with processes elongating along the lines of the pattern. Thus, a protein coating on the diamond layers is highly recommended in situations in which glial cell adhesion to the material is required. H-terminated diamonds should be preferred for protein coating, because surface state measurements indicate that the protein coating is thicker on these substrates than on O-terminated diamonds. However, O-terminated diamonds are much more reproducible than H-terminated diamonds, because H-terminated diamonds are less stable and may rapidly become covered with adsorbed species in contact with ambient air, thereby losing their H termination behavior [35], [36], [37], [38], [39]. This lack of stability may explain the greater variability observed with cultures on H-terminated diamonds. Immediate coating may therefore be required, just after the growth of the material. These properties should be considered when using diamond in implanted stimulating electrodes.

Embryonic neuronal cultures survive well on dispersed nanodiamond particles, indicating that such particles may constitute a potential alternative to protein coating [19]. However, it would be difficult to use these particles alone for implant coating, because they are not covalently bound to the substrate and therefore might be engulfed by phagocytes and transported to other tissues. However, the growth of embryonic neuronal cultures on these dispersed nanodiamond particles [19] suggests that chemical or physical modifications to diamond surfaces during particle preparation may modify cellular interactions with glial cells. Further studies are therefore required to determine the effects of chemical and physical modifications of the diamond on the adhesion and growth of glial cells. An understanding of this glial cell-diamond interface is also important because many neuroprostheses are currently apposed to the glial limiting membrane of the neuronal tissue. This is the case for both subretinal and epiretinal implants, and for visual cortical implants [2], [3], [4]. For such surface implants, increasing glial cell adhesion to the implant and electrodes may become a key issue because it could reduce the electrode/tissue distance and thus increase the stimulation efficacy. In the case of implants with penetrating electrodes, the tissue limiting barrier is ruptured by the penetrating electrodes. Therefore gliosis is important to restore the continuity of the tissue barrier to maintain the tissue homeostasis. The presence of a protein coated diamond at the base of such penetrating needles would allow a rapid sealing of the holes created by the penetrating electrodes. The continuity in the tissue limiting barrier would be reconstituted, preventing any tissue change due to changes in ionic homeostasis. This sealing at the base of the penetrating electrode could leave their tips free of glial cell for the direct neuron/electrode interface. Our results suggest that protein-coated diamond, and protein-coated substrates in general, would be preferable for the improvement of the glial cell-implant interface.

This study not only confirms the biocompatibility of diamond for neurons and glial cells, it also highlights the clear need for protein-coating for some types of expected diamond/cell interface. It therefore supports the consideration of diamond as an attractive electrode material for medical devices interfacing with neural tissues.
